# Supplementary figures and images for: Evaluation of therapeutic efficacy of baloxavir marboxil against high pathogenicity avian influenza virus infection in duck model
Source: PLoS One. 2026 Apr 15;21(4):e0347205. doi: 10.1371/journal.pone.0347205 (PMC13082629; doi:10.1371/journal.pone.0347205)

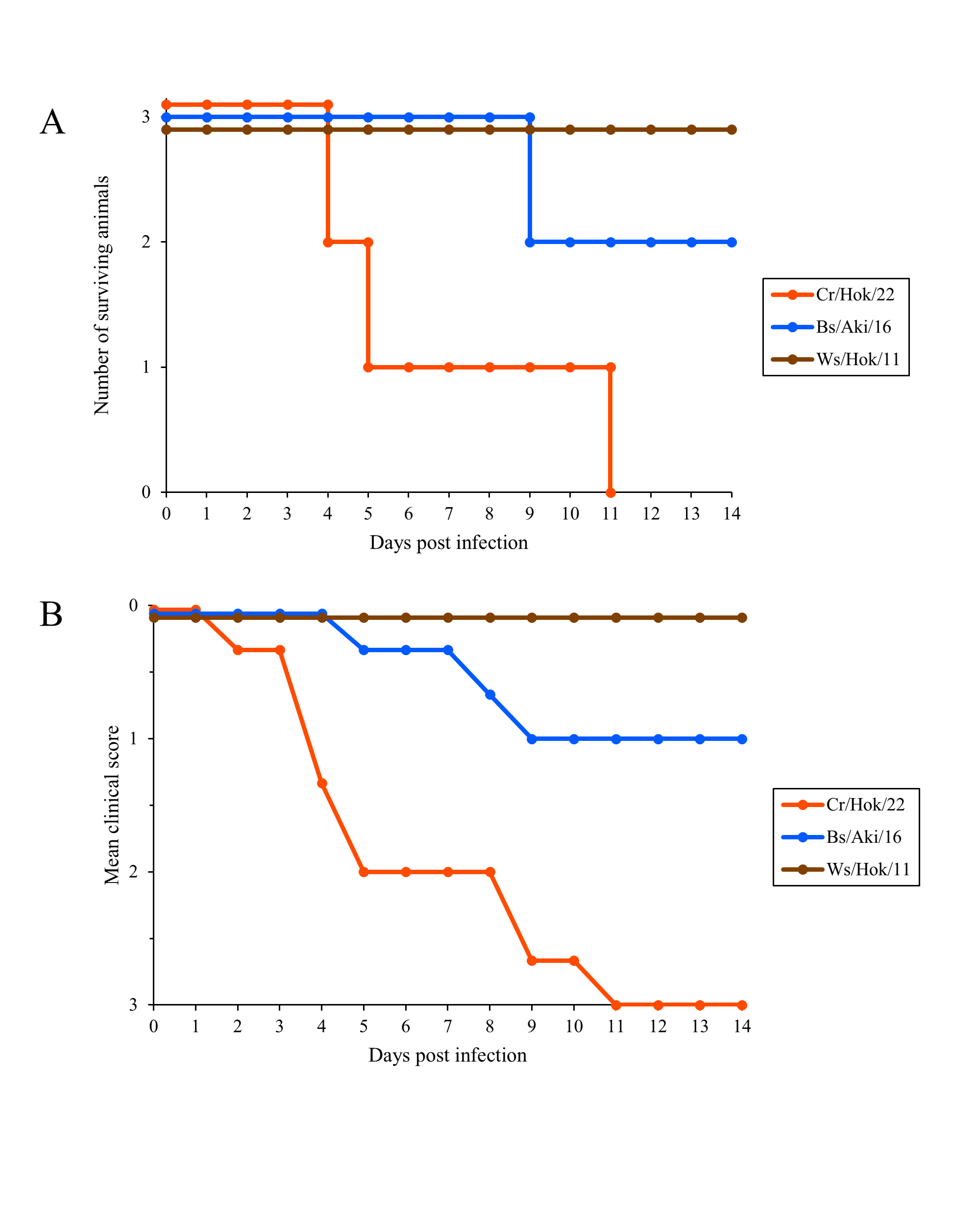

Supplement: S1 Fig — (TIF) [file pone.0347205.s001.TIF]
